# Chromatin resetting mechanisms preventing transgenerational inheritance of epigenetic states

**DOI:** 10.3389/fpls.2015.00380

**Published:** 2015-05-27

**Authors:** Mayumi Iwasaki

**Affiliations:** The Sainsbury Laboratory, University of Cambridge, Cambridge, UK

**Keywords:** transgenerational epigenetic memory, chromatin regulation, vernalization, FLC, ELF6, DDM1, MOM1

## Abstract

Epigenetic regulation can be altered by environmental cues including abiotic and biotic stresses. In most cases, environmentally-induced epigenetic changes are transient, but in some cases they are maintained for extensive periods of time and may even be transmitted to the next generation. However, the underlying mechanisms of transgenerational transmission of environmentally-induced epigenetic states remain largely unknown. Such traits can be adaptive, but also can have negative consequences if the parentally inherited epigenetic memory interferes with canonical environmental responses of the progeny. This review highlights recent insights into the mechanisms preventing transgenerational transmission of environmentally-induced epigenetic states in plants, which resemble those of germline reprogramming in mammals.

## Introduction

Epigenetic marks such as DNA methylation, histone modifications, or histone variants influence the chromatin structure and transcriptional states. These epigenetic marks can be stably maintained, but also can be dynamically altered during development or in response to environmental stimuli.

In most cases, environmentally-induced epigenetic changes are transient. However, in some cases they are stably maintained trough mitotic cell divisions and therefore can be regarded as a type of long-term cellular memory. The best understood example of such epigenetic memory in plants is that of vernalization, which involves the epigenetic silencing of *FLOWERING LOCUS C* (*FLC*) gene by prolonged cold (Kim and Sung, 2012; Song et al., 2012). During embryogenesis, the *FLC* epigenetic state is reset thus allowing the next generation to respond to vernalization signals (further described below).

Epigenetic changes can be meiotically inherited (i.e., transgenerationally transmitted). The inheritance of epigenetic changes is especially well documented in plants where DNA methylation is stably maintained mitotically and meiotically (Law and Jacobsen, 2010). In *Arabidopsis*, DNA METHYLTRANSFERASE1 (MET1) and chromatin remodeling factor DECREASE IN DNA METHYLATION 1 (DDM1) are essential for global maintenance of DNA methylation as shown by the whole genome hypomethylation occurring in *met1* and *ddm1* mutants (Finnegan and Dennis, 1993; Vongs et al., 1993; Jeddeloh et al., 1998; Saze et al., 2003). Many of these hypomethylated loci are stably inherited for many generations even after reintroduction of a functional *MET1* or *DDM1* allele (Johannes et al., 2009; Reinders et al., 2009). Furthermore, transgenes, viral infection, or specific plant tissue culture conditions can also alter DNA methylation patterns in some genes, thus inducing so-called “epialleles” (Vaucheret et al., 1998; Baulcombe, 1999; Vaucheret and Fagard, 2001; Krizova et al., 2009; Rhee et al., 2010).

Besides experimentally-induced epialleles, there are many examples of naturally occurring epialleles inducing visible phenotypes such as flower shape/color, sex determination and genetic incompatibility (Cubas et al., 1999; Iida et al., 2004; Martin et al., 2009; Durand et al., 2012). All natural epialleles reported so far involve changes in DNA methylation. The differences in DNA methylation in the natural epialleles are often associated with transposable elements (TEs) or TE-related sequences located near the genes forming epialleles, suggesting that TE-derived *cis*-regulatory elements contribute to epiallele formation (Weigel and Colot, 2012). TEs are major components of most eukaryotic genomes, and usually silenced with repressive chromatin marks, which are considered a defense mechanism against TE activity since TE transpositions are frequently deleterious to the host. In some cases these epigenetic marks spread to neighboring genes thus altering their expression (Ahmed et al., 2011). Thus, TEs can contribute to epiallele formation.

Interestingly, TEs can be activated transcriptionally and transpositionally by stress in a wild type genetic background. McClintock (1984) suggested that TE activation could be a genomic response to challenge. In support of this view, several reports have described examples of TEs playing roles in gene regulation and genome evolution (Slotkin and Martienssen, 2007; Fedoroff, 2012).

Experimental induction of epialleles and TE mobilization in epigenetic mutants leading to heritable genetic changes has been well documented. However, the occurrence of stable inheritance induced by environmentally induced epigenetic changes has met some controversy (Boyko and Kovalchuk, 2011; Mirouze and Paszkowski, 2011; Paszkowski and Grossniklaus, 2011; Pecinka and Mittelsten Scheid, 2012). The inheritance of environmentally induced-epigenetic changes could be adaptive, but also could be deleterious given that environmental epigenetic memory of parent might impair canonical responses in the progeny.

Recent studies approached the issue from a different perspective, and described the mechanisms preventing transgenerational inheritance of environmentally-induced epigenetic traits.

In this review, I summarize these findings and discuss their implications on the inheritance of environmentally-induced epigenetic changes.

## Resetting Vernalized State

**FIGURE 1:**
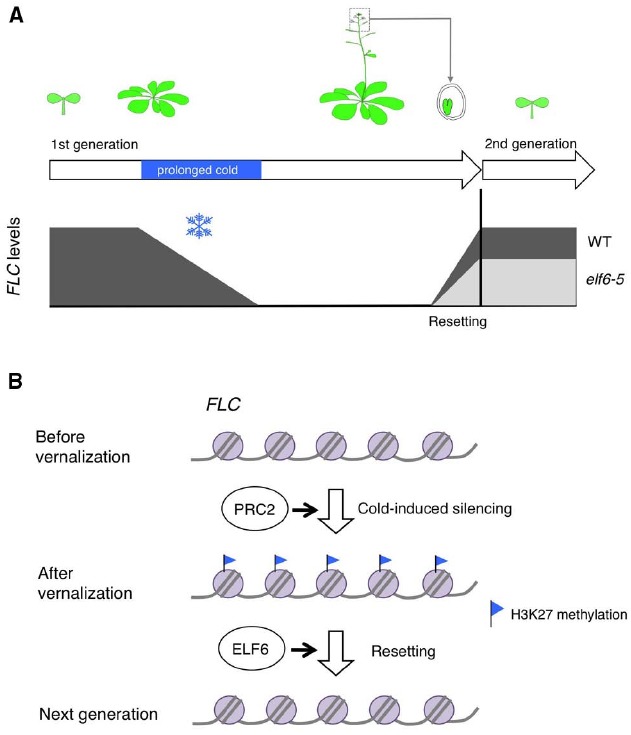
**(A)** Schematic representation of the experimental data in Crevillén et al. (2014). *FLC* is epigenetically silenced by prolonged exposure to cold. Thereafter, the silenced epigenetic state of *FLC* is stably maintained until embryogenesis. In developing embryos the epigenetic state of *FLC* is reset, thus allowing vernalization responses during the new vegetative phase. In the *elf6* hypomorphic-resetting mutant (*elf6-5*), resetting of *FLC* is impaired therefore a partially vernalized state is inherited. **(B)** Simplified model for *FLC* resetting. This silencing of *FLC* is associated with H3K27 methylation mediated by PRC2. Removal of H3K27 methylation by ELF6 is required for restoring the pre-vernalization chromatin state of *FLC*.

Vernalization is the acquisition of ability to flower by exposure of plants with prolonged cold. In *Arabidopsis*, vernalization involves epigenetic silencing of the floral repressor *FLC*, which encodes a MADS box transcription factor (Michaels and Amasino, 1999; Sheldon et al., 1999). *FLC* is expressed throughout the early vegetative development in vernalization-requiring *Arabidopsis* accessions. In response to prolonged cold, *FLC* is epigenetically silenced allowing flowering to be promoted according to other environmental cues such as photoperiod (Figure 1A). This silencing of *FLC* is associated with chromatin modifications including increased levels of H3K27me3 at the *FLC* locus, which is mediated by polycomb repressive complex 2 (PRC2; Bastow et al., 2004; De Lucia et al., 2008; Figure 1B). After the cold exposure, the silenced epigenetic state of *FLC* is stably maintained throughout the rest of the life of the plant until the *FLC* chromatin state is reset during embryogenesis thus reestablishing vernalization requirement to promote flowering in the progeny (Sheldon et al., 2008; Choi et al., 2009; Figure 1A). Whereas the mechanisms leading to *FLC* silencing in response to vernalization have been extensively studied, the mechanisms responsible for resetting *FLC* was less understood.

Recently, Crevillén et al. (2014) screened for mutants in which resetting of *FLC* is impaired so that the vernalized states is inherited in the next generation. This led to the discovery of the histone demethylase EARLY FLOWERING 6 (ELF6) as a component required for *FLC* resetting (Crevillén et al., 2014). In the *elf6* hypomorphic mutants, the progeny from vernalized plants flowered earlier and had reduced *FLC* expression compared to that of the progeny from non-vernalized plants, indicating that vernalized states were transmitted to the *elf6* mutant progeny (Figure 1A).

ELF6 is a jumonji-C-domain-containing protein, and has H3K27me3 demethylase activity. In the *elf6* hypomorphic resetting mutant, an alanine was substituted with a valine in conserved residues of the jumonji C domain, which leads to a reduction in demethylase activity. ChIP analysis showed the H3K27me3 levels were higher in the progeny of vernalized plants than the progeny of non-vernalized plants in the mutants, suggesting that removal of H3K27me3 by ELF6 is required for restoring the pre-vernalization chromatin state associated with *FLC* (Figure 1B). Intriguingly, different *ELF6* alleles are associated with distinct phenotypic responses. Loss-of-function *elf6* alleles are early flowering irrespective of the occurrence of vernalization due to the increased expression of the flowering regulator gene *FT* (Noh et al., 2004). Thus, it was suggested that ELF6 has a broader function, and that the particular hypomorphic mutation reveals a specific aspect of ELF6’s activity to restore the pre-vernalization chromatin state of *FLC* during embryogenesis.

In mammals, reprogramming of epigenetic marks, including H3K27me3, occurs in germ cells and early embryo (Cantone and Fisher, 2013). The reprogramming mediated by ELF6 would be a relevant mechanism conserved in evolution. In *Arabidopsis* genome, H3K27me3 is found in 15% of all genes (Zhang et al., 2007). It would be interesting to address whether other genes are reprogrammed by ELF6 or related proteins.

## Resetting Chromatin Changes Induced by Heat Stress

In 2010, three independent research groups reported the influence of environmental stresses on epigenetically silenced loci in *Arabidopsis* (Lang-Mladek et al., 2010; Pecinka et al., 2010; Tittel-Elmer et al., 2010). They exposed plants to various stress conditions such as temperature shift, drought, elevated salinity, or UV radiation, and examined activities of transcriptionally silenced reporter genes. It was found that heat stress (37 or 42°C) or UV-B radiation releases silencing and activates reporter genes. The release of transcriptional silencing induced by stress occurs at various endogenous loci (Tittel-Elmer et al., 2010). However, this activation is transient since the loci are re-silenced within a few days after stress (Lang-Mladek et al., 2010; Pecinka et al., 2010; Tittel-Elmer et al., 2010). The rapid re-silencing appears to involve nucleosome loading since it is delayed in mutants with impaired chromatin assembly (Pecinka et al., 2010). These results suggest that chromatin non-permissive to transcription displays plasticity in response to stress, but also that there is a robust buffering system that resets chromatin changes to the initial ground state. Interestingly, stress-induced transcriptional activation occurs in differentiated tissues but not in meristematic tissues, suggesting the existence of a mechanism protecting germline cells from epigenetic damage (Pecinka et al., 2010).

Interestingly, the LTR-type retroelement *ONSEN* was found to behave rather exceptionally in response to heat stress. Unlike other heterochromatic loci destabilized by heat, the transcriptional activation persists for longer periods of time (Pecinka et al., 2010; Tittel-Elmer et al., 2010). The heat stress-induced transcriptional activation is enhanced in siRNA defective mutants, however, eventually *ONSEN* transcripts gradually decay as the plant pursues its growth, and no transpositions can be detected in vegetative tissues. Surprisingly, high frequency of transposition is observed in the progeny of siRNA defective mutants subjected to heat stress, suggesting that the siRNA pathway prevents transgenerational transposition of *ONSEN* (Ito et al., 2011).

**FIGURE 2:**
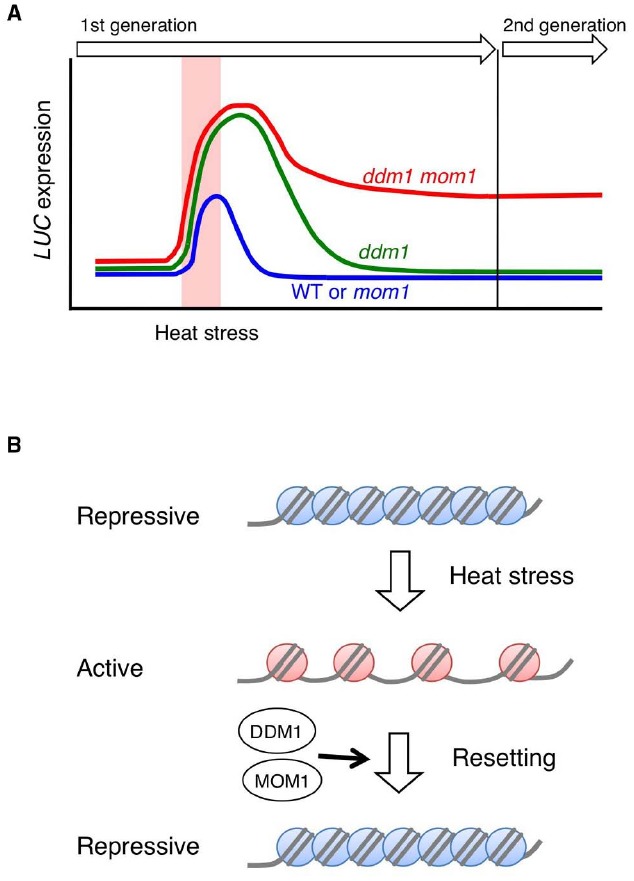
**(A)** Schematic representation of the experimental data in Iwasaki and Paszkowski (2014). A silenced *LUC* transgene is transiently transcriptionally activated in response to a heat stress. In *ddm1* mutants, the heat stress-induced *LUC* activation is stronger and persists longer relative to WT plants. However, the activated state is not transmitted to the progeny. In contrast, in *ddm1 mom1* double mutants, the activation persists in the next generation. **(B)** Schematic illustration heterochromatin states upon heat stress. Heterochromatic loci are transcriptionally activated by heat stress, however, they are rapidly resilenced after stress. DDM1 and MOM1 redundantly reset chromatin states destabilized by heat stress thus preventing transgenerational transmission of transcriptional stress memory.

Recently, Iwasaki and Paszkowski (2014) identified factors preventing transgenerational transmission of stress-induced chromatin changes by forward genetic screen in *Arabidopsis*. A silenced luciferase (*LUC*) reporter gene, whose transcription is transiently activated by heat stress, was used to isolate mutants that retain high or prolonged *LUC* activity after heat stress. This led to the identification of the epigenetic regulators DDM1 and MORPHEUS’ MOLECULE1 (MOM1) as components of a mechanism resetting stress-induced chromatin changes. In the *ddm1* mutant, the heat stress-induced *LUC* activation is stronger and persists longer than WT, but the activated state is not transmitted to the progeny. In the *mom1* mutant, stress-induced activation and subsequent extinction is similar to that of WT. However, and remarkably, in *ddm1 mom1* double mutants, the activation persists in the next generation (Figure 2A). Genome-wide transcriptional profiles revealed that stress-induced transcriptional alterations at various heterochromatic loci were transmitted to next generation in *ddm1 mom1* double mutants. These results indicate that DDM1 and MOM1 redundantly reset chromatin states destabilized by heat stress in order to prevent transgenerational propagation of transcriptional stress memory (Figure 2B).

Both *DDM1* and *MOM1* are required to maintain transcriptional gene silencing (TGS) since mutations in either genes cause release of silencing of heterochromatic loci (Jeddeloh et al., 1998; Amedeo et al., 2000; Steimer et al., 2000). Although *DDM1* and *MOM1* share a number of common target loci for silencing, the regulation mechanism seems to be different for each gene.

DDM1, which is conserved between plants and animals, is a chromatin remodeling factor of the SWI2/SNF2 family (Jeddeloh et al., 1999; Bourc’his and Bestor, 2002; Tao et al., 2011). *ddm1* mutants show progressive global loss of DNA methylation during inbreeding (Kakutani et al., 1996; Jeddeloh et al., 1998). It has been suggested that DDM1 facilitates access of DNA methyltransferases to histone H1-containing heterochromatin (Zemach et al., 2013).

MOM1 is a plant-specific protein with limited homology to the SWI2/SNF2 family whose function remains poorly understood. Mutations in *MOM1* cause release of TGS without major changes in DNA methylation levels, suggesting that MOM1 exerts its silencing function through pathways that are either independent or downstream of those of DNA methylation (Amedeo et al., 2000; Vaillant et al., 2006). Structural and genetic studies indicate that a conserved domain of MOM1 forms a homodimer, which may provide a binding platform for additional silencing factors (Yokthongwattana et al., 2010; Nishimura et al., 2012).

The transient release of transcriptional silencing induced by heat stress is not associated with significant changes in DNA methylation or histone modifications (Pecinka et al., 2010; Tittel-Elmer et al., 2010). Likewise, high levels of DNA methylation were maintained on the promoter of the *LUC* reporter gene in the progeny of heat stressed *ddm1 mom1* mutants despite the occurrence of high *LUC* expression (Iwasaki and Paszkowski, 2014). This strongly suggests that epigenetic marks other than DNA methylation are transmitted to the next generation in the *ddm1 mom1* mutants. The nature of these epigenetic marks necessary for the persistence of stress induced activation of heterochromatic transcription remains to be elucidated.

In summary, this study revealed a previously unidentified function of DDM1 and MOM1 to reset stress-induced chromatin changes. Future studies should address whether similar mechanisms occur in other species given that DDM1 is conserved in yeast and animals.

## Concluding Remarks

The recent progress in our understanding of the mechanisms preventing transgenerational transmission of environmentally-induced epigenetic states opens new avenues for the study of epigenetic inheritance while raising new questions such as that of redundancy of the system. It was reported that DDM1 and MOM1 act redundantly to reset chromatin destabilized by heat stress. Furthermore, although about 3,000 loci on the *Arabidopsis* genome are activated by heat stress (Tittel-Elmer et al., 2010), only about one-tenth remain active in the progeny of heat stressed *ddm1 mom1* mutants, suggesting that other factors act in parallel in the same silencing pathway (Iwasaki and Paszkowski, 2014). Similarly, in the *elf6* hypomorphic mutants, the vernalized state of *FLC* is partially restored. *FLC* expression in the progeny of vernalized *elf6* hypomorphic mutants is lower than in the non-vernalized plants, but still higher than in fully vernalized plants (Crevillén et al., 2014). Thus these observations suggest that other factors act redundantly to reset *FLC*.

These redundancies, essential to confer robustness to the system, would be crucial to ensure erasure of parental memory in order to permit progeny to respond appropriately to current environmental conditions. They could also account for the difficulty in documenting the occurrence of transgenerational transmission of environmentally induced epigenetic traits.

It remains possible that certain environmentally induced epigenetic changes could be inherited and become adaptive as in the case of some TEs which contributed to genome evolution. Further investigations would clarify the issue.

### Conflict of Interest Statement

The author declares that the research was conducted in the absence of any commercial or financial relationships that could be construed as a potential conflict of interest.
